# Supplementary material for: A cryo-EM structure of KTF1-bound polymerase V transcription elongation complex
Source: Nat Commun. 2023 May 30;14:3118. doi: 10.1038/s41467-023-38619-x (PMC10229537; doi:10.1038/s41467-023-38619-x)
Supplement: Supplementary file 3 — Description of Additional Supplementary Files [file 41467_2023_38619_MOESM3_ESM.pdf]

Description of Additional Supplementary Files

File Name: Supplementary Data 1.
Description: The full mass spectrometry report of affinity purified epitope-tagged Pol V from *A. thaliana* T87 cells.
