# Supplementary material for: A cryo-EM structure of KTF1-bound polymerase V transcription elongation complex
Source: Nat Commun. 2023 May 30;14:3118. doi: 10.1038/s41467-023-38619-x (PMC10229537; doi:10.1038/s41467-023-38619-x)
Supplement: Supplementary file 5 — Reporting Summary [file 41467_2023_38619_MOESM5_ESM.pdf]

nature portfolio

Corresponding author(s): Yu Zhang

Last updated by author(s): Apr 28, 2023

Reporting Summary

Nature Portfolio wishes to improve the reproducibility of the work that we publish. This form provides structure for consistency and transparency in reporting. For further information on Nature Portfolio policies, see our Editorial Policies and the Editorial Policy Checklist.

Statistics

For all statistical analyses, confirm that the following items are present in the figure legend, table legend, main text, or Methods section.

n/a	Confirmed	
☒	☐	The exact sample size (*n*) for each experimental group/condition, given as a discrete number and unit of measurement
☒	☐	A statement on whether measurements were taken from distinct samples or whether the same sample was measured repeatedly
☒	☐	The statistical test(s) used AND whether they are one- or two-sided<br>*Only common tests should be described solely by name; describe more complex techniques in the Methods section.*
☒	☐	A description of all covariates tested
☒	☐	A description of any assumptions or corrections, such as tests of normality and adjustment for multiple comparisons
☒	☐	A full description of the statistical parameters including central tendency (e.g. means) or other basic estimates (e.g. regression coefficient) AND variation (e.g. standard deviation) or associated estimates of uncertainty (e.g. confidence intervals)
☒	☐	For null hypothesis testing, the test statistic (e.g. *F*, *t*, *r*) with confidence intervals, effect sizes, degrees of freedom and *P* value noted<br>*Give P values as exact values whenever suitable.*
☒	☐	For Bayesian analysis, information on the choice of priors and Markov chain Monte Carlo settings
☒	☐	For hierarchical and complex designs, identification of the appropriate level for tests and full reporting of outcomes
☒	☐	Estimates of effect sizes (e.g. Cohen's *d*, Pearson's *r*), indicating how they were calculated

Our web collection on statistics for biologists contains articles on many of the points above.

Software and code

Policy information about availability of computer code

Data collection	The EPU (version 2.12) was used to collect the cryo-EM micrographs.
Data analysis	MotionCor2 , CTFFIND4 , cryoSPARC v3.and RELION 3.1 were used to process cryo-EM single particles.<br>Phenix (version 1.9-1692) was used to perform structure refinement.<br>Coot (version 0.8 .2) was used to perform structure model building.<br>UCSF Chimera (version 1.14), chimeraX (version 0.9) and PyMOL Molecular Graphics System (Version 2.0 Schrodinger, LLC) were used for structure analysis.<br>AlphaFold (version 2.0) was used for structure modeling.<br>Mascot (version 2.2) was used for mass spectrometry analysis.

For manuscripts utilizing custom algorithms or software that are central to the research but not yet described in published literature, software must be made available to editors and reviewers. We strongly encourage code deposition in a community repository (e.g. GitHub). See the Nature Portfolio guidelines for submitting code & software for further information.

Data

Policy information about availability of data

All manuscripts must include a data availability statement. This statement should provide the following information, where applicable:

- Accession codes, unique identifiers, or web links for publicly available datasets
- A description of any restrictions on data availability
- For clinical datasets or third party data, please ensure that the statement adheres to our policy

Coordinates and cryo-EM maps have been deposited in the PDB and EMDB under ID code 8HYJ and EMD-35086. 5XON, 2E2H, 1Y1V, 5IYC, 7EU0, and 5XOG used for structure analysis. The protein mass spectrometry proteomics data have been deposited in ProteomeXchange repository with the accession number PXD041996.

Human research participants

Policy information about studies involving human research participants and Sex and Gender in Research.

Reporting on sex and gender	N/A
Population characteristics	N/A
Recruitment	N/A
Ethics oversight	N/A

Note that full information on the approval of the study protocol must also be provided in the manuscript.

Field-specific reporting

Please select the one below that is the best fit for your research. If you are not sure, read the appropriate sections before making your selection.

☒ Life sciences ☐ Behavioural & social sciences ☐ Ecological, evolutionary & environmental sciences

For a reference copy of the document with all sections, see nature.com/documents/nr-reporting-summary-flat.pdf

Life sciences study design

All studies must disclose on these points even when the disclosure is negative.

Sample size	The sample size has been described in the figure legends. No statistical method has been used to predetermine the sample size. Experiments were typically repeated in triplicates independently to ensure the robustness of conclusions.
Data exclusions	No data was excluded.
Replication	The replicate numbers for each experiments were described in the respective figure legends. Experiments were typically repeated in triplicates independently to ensure the robustness of conclusions. All attempts at replication were successful.
Randomization	Not applicable as no groups to be allocated.
Blinding	Not applicable as no groups to be allocated.

Reporting for specific materials, systems and methods

We require information from authors about some types of materials, experimental systems and methods used in many studies. Here, indicate whether each material, system or method listed is relevant to your study. If you are not sure if a list item applies to your research, read the appropriate section before selecting a response.

Materials & experimental systems

n/a	Involved in the study
☒	☐ Antibodies
☐	☒ Eukaryotic cell lines
☒	☐ Palaeontology and archaeology
☒	☐ Animals and other organisms
☒	☐ Clinical data
☒	☐ Dual use research of concern

Methods

n/a	Involved in the study
☒	☐ ChIP-seq
☒	☐ Flow cytometry
☒	☐ MRI-based neuroimaging

Eukaryotic cell lines

Policy information about cell lines and Sex and Gender in Research

Cell line source(s)	The Arabidopsis thaliana T87 cells.
Authentication	The cells were used in protein complex preparation and authentication was not required.
Mycoplasma contamination	The cell line was not tested for Mycoplasma contamination.
Commonly misidentified lines (See ICLAC register)	No commonly misidentified lines were used.
